# The Genetic Architecture of Congenital Heart Disease in Neonatal Intensive Care Unit Patients—The Experience of University Medical Centre, Ljubljana

**DOI:** 10.3390/life14091118

**Published:** 2024-09-05

**Authors:** Ana Peterlin, Sara Bertok, Karin Writzl, Luca Lovrečić, Aleš Maver, Borut Peterlin, Maruša Debeljak, Gregor Nosan

**Affiliations:** 1 Institute of Histology and Embryology, Faculty of Medicine, University of Ljubljana, 1000 Ljubljana, Slovenia; 2 Clinical Institute of Genomic Medicine, University Medical Centre Ljubljana, 1000 Ljubljana, Slovenia; karin.writzl@kclj.si (K.W.); luca.lovrecic@kclj.si (L.L.); ales.maver@kclj.si (A.M.); borut.peterlin@kclj.si (B.P.); 3 Department of Genetics, Department of Endocrinology, Diabetes, and Metabolic Diseases, University Children’s Hospital, University Medical Centre Ljubljana, 1000 Ljubljana, Slovenia; sara.bertok@kclj.si; 4 Clinical Institute for Special Laboratory Diagnostics, University Children’s Hospital, University Medical Centre Ljubljana, 1000 Ljubljana, Slovenia; marusa.debeljak@kclj.si; 5 Department of Pediatrics, Faculty of Medicine, University of Ljubljana, 1000 Ljubljana, Slovenia; 6 Department of Neonatology, Division of Paediatrics, University Medical Centre Ljubljana, 1000 Ljubljana, Slovenia; gregor.nosan@kclj.si

**Keywords:** congenital heart disease, chromosomal microarray analysis, next-generation sequencing, diagnostic yield

## Abstract

Congenital heart disease (CHD) is the most commonly detected congenital anomaly and affects up to 1% of all live-born neonates. Current guidelines support the use of chromosomal microarray analysis (CMA) and next-generation sequencing (NGS) as diagnostic approaches to identify genetic causes. The aim of our study was to evaluate the diagnostic yield of CMA and NGS in a cohort of neonates with both isolated and syndromic CHD. The present study included 188 infants under 28 days of age with abnormal echocardiography findings hospitalized at the Department of Neonatology, UMC Ljubljana, between January 2014 and December 2023. Phenotypic data were obtained for each infant via retrospective medical chart review. We established the genetic diagnosis of 22 distinct syndromes in 17% (32/188) of neonates. The most frequent genetic diagnoses in diagnosed cases were *22q11.2* microdeletion and CHARGE syndromes, followed by Noonan syndrome and Williams syndrome. In addition, we detected variants of uncertain significance in 4.8% (9/188) of neonates. Timely genetic diagnosis is important for the detection of syndrome-related comorbidities, prognosis, reproductive genetic risks and, when appropriate, genetic testing of other family members.

## 1. Introduction

Congenital heart defects (CHDs) are structural anomalies of the heart and great vessels that result from errors in early embryogenesis [1]. They are the most commonly detected congenital anomaly, affecting approximately 0.8 of all live-born infants [2], and are a major cause of morbidity and mortality in infancy [3].

The etiology of CHD is multifactorial and involves interplay between genetic and environmental factors. CHDs can be caused by environmental exposure to teratogens such as drugs, viral infections, and maternal conditions such as obesity and diabetes [4]. Genetic influences are supported by the relatively high risk of recurrence in families and the well-established association of CHD with chromosomal abnormalities [5]; however, the cause of the disease remains unexplained in up to 60% of CHD patients [6]. Elucidation of genetic causes is also challenging because of CHD’s genetic and phenotypic heterogeneity [7]. Current diagnostic methods, including cytogenetic techniques (karyotyping and copy number variant platforms) and next-generation sequencing (NGS), can reach a genetic diagnosis in 18–36% of CHD patients [8]. The overall yield of genetic testing depends on the type of congenital heart malformation, the presence of extracardiac congenital anomalies, and the applied genetic test modality [8,9].

The value of genetic testing in the setting of CHD lies in defining etiology and consequently ending diagnostic odyssey for patients and families, possible detection of additional health problems associated with genetic diagnosis, prognostic information for clinical outcomes, genetic reproductive risks for the family, and genetic testing of additional family members when appropriate [10,11].

This study aimed to assess the diagnostic yield of genetic testing in the clinical evaluation of neonates with diagnosed CHD who were hospitalized at the Department of Neonatology, Division of Paediatrics, University Medical Centre (UMC), Ljubljana.

## 2. Materials and Methods

### 2.1. Patient Selection

In the present study, we enrolled neonates aged <28 days with abnormal echocardiographic findings who were hospitalized in the Department of Neonatology, Division of Paediatrics, UMC Ljubljana between January 2014 and December 2023. Clinical characteristics were obtained through a retrospective chart review of each neonate. Neonates with isolated hemodynamically insignificant patent ductus arteriosus (PDA), isolated hemodynamically insignificant patent foramen ovale (PFO), cardiomyopathy, vasculopathy, and exposure to known environmental teratogenic factors were excluded. We also excluded neonates with prenatally or perinatally detected common trisomies, namely trisomies 13, 18, and 21. Neonates were assigned to one of the two groups based on the presence of isolated CHD or additional extracardiac anomalies. Neonates with isolated CHD were subdivided into three groups based on the complexity of congenital heart malformations according to the Botto classification (simple, association, and complex) [12]. Chromosomal microarray analysis (CMA) was performed as a first-tier genetic test in all neonates with CHD, except for 11 neonatal patients in whom a high possibility of monogenic disorder based on the clinical presentation was detected, and next-generation sequencing (NGS) was performed as a first-tier genetic test instead. However, in most neonatal CHD patients, NGS was performed in neonates with normal CMA results and clinical suspicion of monogenic disease. Informed consent was obtained from the parents of the neonates, in accordance with the guidelines established by the institutional review boards at their primary site of care.

### 2.2. Genetic and Bioinformatic Analysis

#### 2.2.1. CMA and Classification of Results

DNA was isolated from peripheral blood samples using the Qiagen Mini Kit (Qiagen, Valencia, CA, USA) according to the manufacturer’s protocol. The quality and concentration parameters of the DNA were measured using a NanoDrop 2000c spectrophotometer (Thermo Fisher Scientific Inc., Waltham, MA, USA) and a Qubit 2.0 fluorometer (Life Technologies Inc., Waltham, MA, USA). Following sample extraction, the DNA was processed according to the Agilent protocol (Version 8.0 December 2019) using commercially available male and female genomic DNA (Agilent Technologies, Santa Clara, CA, USA, human reference DNA, male and female) as reference DNA. Agilent SurePrint G3 Unrestricted CGH 4 × 180 K microarrays were used, which provided a practical average resolution of 50 kb. Array images were acquired using an Agilent laser scanner G2565CA. The image files were quantified using the Agilent Feature extraction software for Cytogenomics 5.3, and analyzed with the Agilent Cytogenomics 5.3 software (Agilent Technologies). Called copy number variants (CNV) were aligned with known aberrations in publicly available databases, ClinGen (https://clinicalgenome.org/, accessed on 19 July 2024), DECIPHER (Database of Chromosomal Imbalance and Phenotype in Humans using Ensembl Resources (https://www.deciphergenomics.org/, accessed on 19 July 2024), and the Database of Genomic Variants (DGV) (http://dgv.tcag.ca/dgv/app/home, accessed on 19 July 2024), as well as with the in-house database of detected variants and their clinical significance, ascertained by trained analysts. All called CNVs were classified according to ACMG Standards and Guidelines [13].

#### 2.2.2. Next-Generation Sequencing and Variant Interpretation

NGS was performed on isolated DNA samples at the Clinical Institute for Special Laboratory Diagnostics, University Children’s Hospital, UMC Ljubljana, and/or the Clinical Institute of Genomic Medicine, UMC Ljubljana.

At the Clinical Institute of Genomic Medicine, UMC Ljubljana, fragmentation and enrichment of the isolated DNA samples were performed according to the protocol Twist CORE Exome or Nextera Coding Exome, followed by sequencing on an Illumina NovaSeq 6000 (Cegat, Tübingen, Germany) or Illumina NextSeq 550 (UMCL, Ljubljana, Slovenia) in 2 × 150 cycles or 2 × 100 cycles, respectively. To process the sequencing data, we utilized an in-house developed workflow defined in the WDL language (workflow definition language). The versioned and updated source code of the complete workflow is available at the following GitHub repository (https://github.com/AlesMaver/CMGpipeline, accessed on 19 July 2024). Briefly, after duplicates were removed, the alignment of reads to the GRCh38 reference assembly was performed using the BWA algorithm (v0.6.3), and variant calling was performed using the GATK framework (v2.8). Only variants exceeding the quality score of 30.0 and depth of 5 were used for downstream analyses. Variant annotation was performed using ANNOVAR and snpEff algorithms with pathogenicity predictions in the dbNSFPv2 database. The reference gene models and transcript sequences were obtained from the RefSeq database (https://www.ncbi.nlm.nih.gov/refseq/, accessed on 19 July 2024). Structural variants were assessed using the CONIFER v0.2.2 algorithm. Variants with a population frequency exceeding 1% in gnomAD (https://gnomad.broadinstitute.org/, accessed on 19 July 2024), synonymous variants, intronic variants, and variants outside of the clinical target were filtered out during the analyses. The interpretation of sequence variants was based on ACMG/AMP standards and guidelines [14].

NGS library preparation was performed according to standard Illumina protocols (Illumina DNA Prep with Enrichment) at the Clinical Institute for Special Laboratory Diagnostics, University Children’s Hospital, UMC Ljubljana). WES sequences were generated using the Illumina NovaSeq 6000 system. A bcbio-nextgen workflow toolkit (https://bcbio-nextgen.readthedocs.io/, accessed on 19 July 2024) was used for bioinformatics analyses. Reads were aligned to the GRCh38 assembly of the human genome with BWA-mem [15] using samtools and sambamba [16] to sort bam files and mark duplicate reads. Variant calling was performed according to GATK Best Practices Workflows for small germline variants calling with HaplotypeCaller [17]. VarAFT software version 2.x was used to annotate and filter identified genetic variants with coverage >10× and read frequency >0.3 [18]. Copy number variations in the region of interest (ROI) were inferred using the CNVkit Python library [19]. The minor allele frequency threshold for known variants was set at 1%, and all variants exceeding this value were excluded from further analysis. All variants were classified according to the American College of Medical Genetics and Genomics/Association for Molecular Pathology (ACMG/AMP) variant pathogenicity guidelines [14].

### 2.3. Statistics

We analyzed whether there was a statistically significant difference in the establishment of a genetic diagnosis for neonatal patients with isolated CHD and patients who presented with extracardiac anomalies in addition to CHD. The results were considered statistically significant at *p* ≤ 0.01. Statistical analyses were performed using IBM SPSS Statistics (Version 26).

## 3. Results

**Figure 1:**
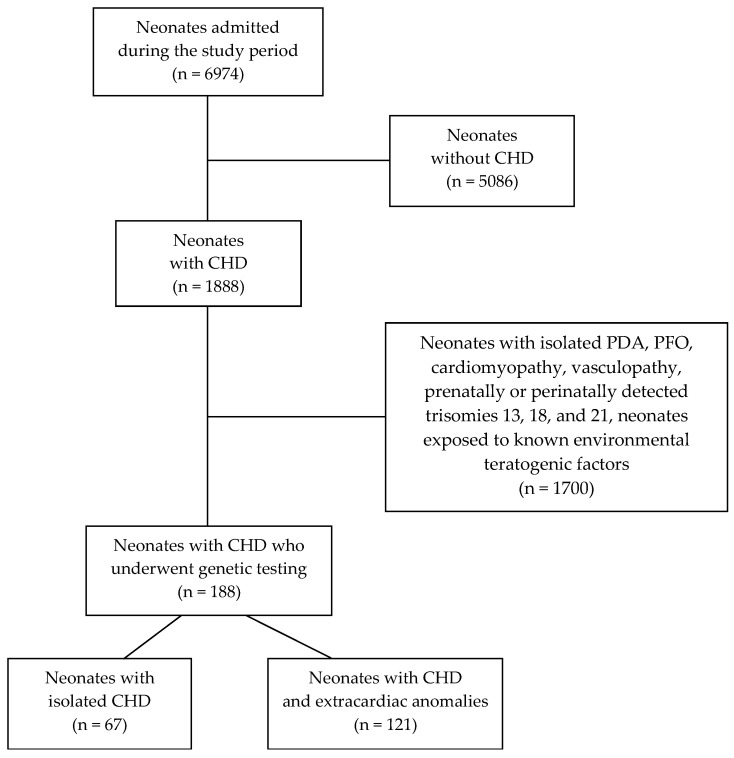
CONSORT diagram detailing neonatal CHD patient selection.

**Figure 2:**
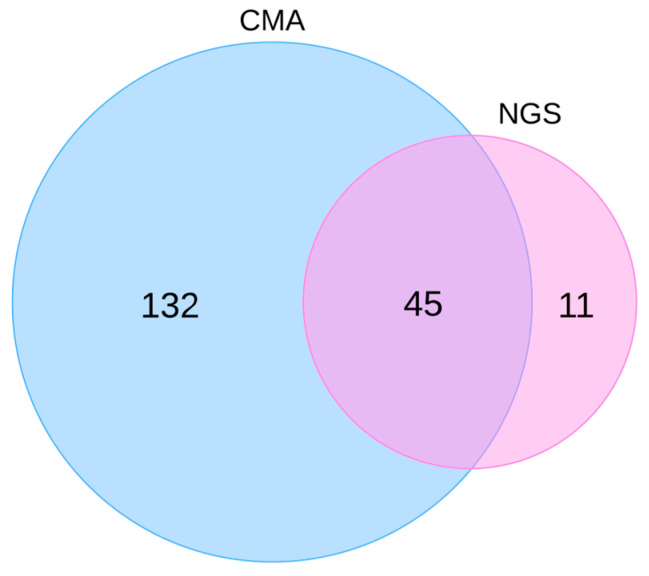
Venn diagram showing the distribution of genetic testing modalities applied in the cohort of neonates with CHD.

This study included 188 neonates diagnosed with CHD who underwent genetic testing when hospitalized at the Department of Neonatology, Division of Paediatrics, UMC Ljubljana. The cohort comprised 36% (67/188) of the neonates with isolated CHD and 64% (121/188) of the neonates with CHD and additional extracardiac congenital anomalies (Figure 1). Neonates clinically diagnosed with isolated CHD were assigned to one of three groups according to the Botto classification: 15% (29/188) of neonates were diagnosed with simple isolated CHD, 10% (18/188) with an association, and 11% (20/188) with complex CHD (Table 1). CMA was performed as a first-tier test in 94.1% (177/188) of neonates. In 5.9% (11/188) of neonates, NGS was performed instead because of the characteristic clinical presentation specific for a monogenic genetic cause, while in 45 neonates, the NGS was performed after the negative CMA result (Figure 2). In the two cases with abnormal CMA results, karyotyping and FISH were employed to further delineate chromosomal aberrations.

We established a genetic diagnosis for 22 distinct genetic syndromes in 17% (32/188) of the neonates. Genetic causes of CHD were identified in 24.8% (30/121) of neonates with CHD and additional extracardiac anomalies and 3% (2/67) of neonates with isolated CHD. Detection of additional extracardiac anomalies was associated with a statistically significant rate for the establishment of genetic diagnosis (chi-square = 9.65, *p* = 0.002). In this group, 111 patients had CMA and 47 had NGS, while all 67 patients had CMA, and 9 had NGS in the isolated CHD group. For neonates with isolated CHD, the diagnosis was made in 5% (1/20) of complex isolated CHD patients and 5.6% (1/18) of association patients and none of the patients diagnosed with simple isolated CHD. The diagnosis was reached by CMA in 10.1% (19/188) of the neonates. The most common microdeletion syndromes were 22q11.2 microdeletion syndrome (15.6%; 5/32), and Williams syndrome (6.2%; 2/32) (Table 2). Using NGS either sequentially after CMA or as a first-tier genetic test, 6.9% (13/188) of neonates with CHD were diagnosed. The most frequent monogenic conditions identified were CHARGE syndrome (15.6%, 5/32) and Noonan syndrome (6.2%, 2/32) (Table 2).

In one patient, we established a dual genetic diagnosis of 17q12 microduplication syndrome and Weaver syndrome. Their clinical presentation was a combination of signs and symptoms characteristic for both conditions. Clinical characteristics and genetic diagnoses are described in detail in Table 2 and Table 3. Variants of uncertain significance, detected in 4.8% (9/188) of the patients, are presented in Table 4. All sequence variants detected by NGS were absent from the GnomAD v.4.1.0 (https://gnomad.broadinstitute.org/, accessed on 19 July 2024).

The identification of a genetic diagnosis led to a change in the medical management of all 32 patients for whom the genetic aetiology of their condition was discerned. Namely, referrals tailored to the identified genetic conditions of diagnosed patients were initiated, accompanied by the planning of comprehensive surveillance to address health risks associated with specific genetic diseases. Additionally, parents were directed to the Genetic Outpatient Clinic for counselling regarding the risk of recurrence and available family planning options.

## 4. Discussion

We identified a genetic cause in 17% of the patients in a cohort of 188 neonates with CHD. As clinical characteristics of genetic diseases are not always fully present at birth but may only become apparent during the course of the child’s development (e.g., global developmental delay), neonatal CHD patients present a diagnostic challenge that differs from that of pediatric or adult patients. The general recommendation for clinical genetic testing in CHD includes CMA as a first-tier test and exome sequencing as a second-tier genetic test [20,21]. In this paper, we report the clinical experience of using the recommended protocol in a cohort of neonates in the Slovenian National Tertiary Centre.

In the present study, the overall diagnostic yield of CMA was 10.1%. The reported diagnostic yields in other studies varied considerably among the different CHD subgroups. For example, in a prenatal cohort of 147 fetuses that underwent genetic testing due to the presence of CHD, a genetic diagnosis was obtained by CMA in 13.7% of cases [22]. Unsurprisingly, studies that included only patients with syndromic presentation reported higher diagnostic yields in the range of 20–50% [23,24,25].

Exome and whole-genome sequencing are increasingly used in research, but also in the clinical setting. The incremental yield of whole-genome sequencing over QF-PCR and CMA was estimated to be 26% for a cohort of patients with congenital anomalies, with no significant increase in yield compared with exome sequencing. [26]. Another study demonstrated a diagnostic yield of 27% with rapid WGS in individuals with CHD, leading to changes in clinical management in 62% of the patients with diagnostic results [27]. However, diagnostic rates still differ across studies and among the tested subgroups of congenital heart disease. Statistically significant higher diagnostic frequencies of positive genetic findings were continuously observed in patients with syndromic CHD in our study as well as in other similar studies [28,29,30].

Interestingly, a dual genetic diagnosis was established in one patient in our cohort, with a combination of clinical signs of both 17q12 microduplication syndrome and Weaver syndrome, highlighting the complexity of making a genetic diagnosis.

We found a genetic diagnosis in 3% of the neonates with isolated CHD. It was estimated that 13.4% of infants with isolated CHD with identifiable genetic causes would have been missed if genetic testing had not been offered [31]. Although the yield of genetic testing in newborns with isolated CHD is relatively low, it is still important to offer genetic testing because of the clear clinical benefit of molecular diagnosis. Timely genetic diagnosis in the Neonatal Intensive Care Unit (NICU) setting presents potential for enhancing management strategies. For a specific subset of patients, this diagnosis may provide an opportunity to access targeted or experimental treatment for rare diseases. Conversely, for many patients, diagnosis, contingent upon the severity of the genetic condition and its prognosis, may lead to a decrease in invasive diagnostic procedures, implementation of tailored management plans, and surveillance for complications, potentially resulting in improved long-term outcomes. In cases where the prognosis is extremely poor, particularly in situations involving profoundly debilitating or life-threatening conditions, diagnosis may prompt earlier discussions regarding palliative care. A genetic diagnosis also concludes the traditionally long and often invasive diagnostic process for both parents and clinicians. Moreover, precise genetic diagnosis enables families to make informed decisions about future reproductive choices, even when such information does not directly affect the clinical care of the neonates [32,33].

## 5. Conclusions

In this study, we described the molecular genetic pathology of CHD in the Slovenian population and highlighted the importance of comprehensive genetic analysis of CHD. Timely genetic diagnosis is important for the detection of syndrome-related comorbidities, prognosis, reproductive genetic risks, and predictive genetic testing of at-risk family members. Systematic implementation of new genetic testing approaches, including whole-genome sequencing, optical genome mapping, and long-read sequencing, might improve the diagnostic yield in the future.

## Figures and Tables

**Table 1:** Patients divided into groups according to the Botto classification of congenital heart defects (CHDs).

Category		N of Neonates (%)	N of Neonates CMA (%)	N of Neonates NGS (%)
Isolated CHD	Simple	29 (15)	29 (100%)	5 (17.2%)
	Association	18 (10)	18 (100%)	0
	Complex	20 (11)	20 (100%)	4 (20%)
CHD with extracardiac defect		121 (64)	111 (91.7%)	47 (38.8%)

**Table 2:** Neonates with congenital heart disease and detected disease-causing copy number variants.

N	Congenital Heart Disease	Extracardiac Defects	Botto Classification	Type of Genetic Test	Results of Genetic Diagnostics	Genetic Classification	Syndrome
1	sASD	kidney anomaly	MCA	CMA	arr[hg38] 7q11.23(73,352,304–74,719,013)×1	P	Williams syndrome
2	SVAS + stenosis of both pulmonary arteries	/	Isolated, association	CMA	arr[hg38] 7q11.23(74,060,601–74,079,563)×1	P	Williams syndrome
3	mVSD, BAV	hypotonia, hypoplasia of the corpus callosum, feeding difficulties, cryptorchidism, dysmorphic facies	MCA	CMA	46,XY, del(8)(p23.3p23.3),dup(8)(p12p23)dn	P	8p inverted duplication/deletion syndrome
4	VSD	congenital hydronephrosis, dysmorphic facies	MCA	CMA	arr[GRCh38] 10q26.13q26.3(124,840,258–133,247,600)×1	P	10q26 deletion syndrome
5	VSD, ASD	dysmorphic facies	MCA	CMA	arr[GRCh38] 22q11.21(20,726,972–21,076,885)×1	P	22q11.2 microdeletion syndrome
6	pmVSD	coloboma of irises, hypotonia, anorectal anomaly, feeding difficulty	MCA	CMA	arr[GRCh38]11q23.3q25(1,193,69473–134,904,063)×1	P	Jacobsen syndrome
7	TGA, ASD, PDA	LGA	MCA	CMA+NGS	arr[GRCh38] 17q12(36,792,631–37,854,407)×3, mat	P	17q12 microduplication syndrome
					*EZH2*(NM_004456.5): c.2051G>A	P	Weaver syndrome
8	aortic valve stenosis, BAV, sASD	/	Isolated, complex	CMA	47,XY,+mar.ish der(22)(pter->q11.21::p12->pter)(acro-p++,SE14/22+,CEP22+,N25+)	P	Cat eye syndrome
9	ToF, ASD	dysmorphic facies	MCA	CMA	arr[GRCh38] 1q21.1q21.2(147,147,409–143,729,392)×3 dn	P	1q21.1 microduplication syndrome
10	VSD	hypocalcemia, dysmorphic facies	MCA	CMA	arr[GRCh38] 22q11.21(18,925,357–21,076,885)×1 dn	P	22q11.2 microdeletion syndrome
11	pmVSD, multiple ASDs, PFO	SGA, palatoschisis, dysmorphic facies, proximal placement of thumb, pes calcaneovalgus	MCA	CMA	arr[GRCh38]18q21.31q23(57,444,618–80,244,381)×1	P	18q deletion syndrome
12	VSD, ASD	renal cysts	MCA	CMA	arr[GRCh38] 17p11.2(16,938,849–20,314,464)×1	P	Smith–Magenis syndrome
13	pmVSD, truncus arteriosus,	hypothyroidism	MCA	CMA	arr[GRCh38] 22q11.21(18,930,283–21,076,885)×1	P	22q11.2 microdeletion syndrome
14	VSD, ASD	dysmorphic facies	MCA	CMA	arr[GRCh38] 22q11.21(18,930,283–21,076,885)×1	P	22q11.2 microdeletion syndrome
15	VSD, ASD	dysmorphic facies	MCA	CMA	arr[GRCh38] 1q21.1q21.2(147,147,409–148,353,946)×1 dn	P	1q21.1 microdeletion syndrome
16	mVSD, sASD, hypoplastic aortic arch	dysmorphic facies	MCA	CMA	arr[GRCh38] 22q11.21(18,930,283–21,076,885)×1	P	22q11.2 microdeletion syndrome
17	ASD, PDA	dysmorphic facies	MCA	CMA	arr[GRCh38] 16p13.11(15,032,852–16,198,378)×3	P	16p13. 11 microdeletion syndrome
18	ASD	hypotonia, hydronephrosis	MCA	CMA	arr[GRCh38] 16p13.11(15,032,852–16,198,378)×3	P	16p13.11 microduplication syndrome
19	stenosis of aortic valve, BAV	dysmorphic features	MCA	CMA	arr(X)×1[0.8]	P	mosaic Turner syndrome

mat—maternally inherited, LGA—large for gestational age, MCA—multiple congenital anomalies, mVSD—muscular VSD, P—pathogenic variant, PDA—patent ductus arteriosus, PFO—patent foramen ovale, pmVSD—perimembranous VSD, sASD—ASD secundum, SVAS—supravalvular aortic stenosis, ToF—tetralogy of Fallot, VSD—ventricular septal defect.

**Table 3:** Neonates with congenital heart disease and detected disease-causing single nucleotide variants.

N	Congenital Heart Disease	Extracardiac Defects	Botto Classification	Type of Genetic Test	Results of Genetic Diagnostics	Genetic Classification	Syndrome
1	ToF	EA/TEF	MCA	CMA+NGS	*CHD7*(NM_017780):c.5405-8C>G	P	CHARGE syndrome
2	valvular pumonary stenosis, SVPS	dysmorphic facies, macrosomia, unilateral cryptorchidism, aplasia cutis	MCA	NGS	*PTPN11*(NM_002834.5):c.923A>G, p.Asn308Ser	P	Noonan syndrome
3	sASD	hypotonia, hypoplasia of the corpus callosum, dysmorphic features, palatoschisis, glossoschissis, hypermobility of joints, clinodactyly of 5th fingers	MCA	NGS	*OFD1*(NM_003611.3):c.1193_1196del, p.Gln398Leufs*2	LP	Orofaciodigital syndrome I
4	AVSD	coloboma of iris, facial nerve palsy, mixed hearing loss, hypotonia dysmorphic features, feeding difficulties	MCA	NGS	*CHD7*(NM_017780.4):c.4353+1G>A	P	CHARGE syndrome
5	sASD, BAV, PDA	dysmorphic facies, palatoschisis, widely spaced nipples, barrel chest, hypermobility of joints, clinodactyly of 5th fingers	MCA	CMA+NGS	*KMT2D*(NM_003482.4):c.4364dup, p.Tyr1455*	P	Kabuki syndrome
6	sASD, cleft mitral valve with mild MVR, PDA	dysmorphic facies, chorioretinal coloboma, vocal cord paresis, feeding difficulties, hearing loss	MCA	NGS	*CHD7*(NM_017780.4):c.3655C>T, p.Arg1219*	P	CHARGE syndrome
7	ToF	brachycephaly, ptosis of right eyelid, coloboma of optic nerve papilla, gnatoschisis, choanal atresia, feeding difficulties, unilateral renal agenesis, dysmorphic features, hockey-stick palmar crease, partial 2–3 toe syndactyly, hypotonia, hearing loss	MCA	CMA+NGS	*CHD7*(NM_017780.3):c.4203_4204delT, p.His1401Glnfs*20	P	CHARGE syndrome
8	sASD, aortic valve stenosis, BAV	AMC, dynamic upper airway obstruction, ptosis of right eyelid, cryptorchidism, bilateral congenital hip dislocation, clubfoot, fibromatosis colli	MCA	NGS	*CHRNG*(NM_005199.5):c.753_754del, p.Val253Alafs*44	P	Multiple pterygium syndrome— Escobar type
					*CHRNG*(NM_005199.5):c.250G>A, p.Asp84Asn	LP	
9	sASD, PPS, PDA	dysmorphic facies, direct hyperbilirubinemia	MCA	NGS	*JAG1*(NM_000214.3):c.2122_2125del, p.Gln708Valfs*34	P	Alagille syndrome
10	pulmonary valve stenosis, PDA, PFO	dysmorphic facies, LGA, renal cyst	MCA	NGS	*PTPN11*(NM_002834.5):c.922A>G, p.Asn308Asp	P	Noonan syndrome
11	pulmonary valve stenosis, BAV, bicuspid pulmonary valve, PFO	dysmorphic facies, bilateral coloboma of iris, macula and papilla, horseshoe kidney, ankyloglossia	MCA	CMA+NGS	*CHD7*(NM_017780.4):c.6292C>T, p.Arg2098*	P	CHARGE syndrome
12	pmVSD	hypotonia, abnormal cortical gyration, feeding difficulties, dysmorphic facies, single palmar crease	MCA	CMA+NGS	*SMARCA4*(NM_003072.5):c.4114C>T, p.Arg1372Cys	LP	Coffin-Siris syndrome 4
13	left atrial isomerism	heterotaxy, polysplenia	MCA	NGS	*DNAAF3*(NM_001256715.2):c.73_82del, p.Leu25Lysfs*20	LP	Ciliary dyskinesia, primary, 2

AMC—arthrogryposis multiplex congenita, EA/TEF—esophageal atresia/tracheoesophageal fistula, LGA—large for gestational age, LP—likely pathogenic variant, MCA—multiple congenital anomalies, MVR—mitral valve regurgitation, mVSD—muscular VSD, P—pathogenic variant, PAH—pulmonary artery hypertension, PA-VSD—pulmonary atresia with ventricular septal defect, PDA—patent ductus arteriosus, PFO—patent foramen ovale, pmVSD—perimembranous VSD, PPS—peripheral pulmonary stenosis, sASD—ASD secundum, SGA—small for gestational age, SVAS—supravalvular aortic stenosis, ToF—tetralogy of Fallot, VSD—ventricular septal defect.

**Table 4:** Neonates with congenital heart disease and detected copy number variants or single nucleotide variants of uncertain significance (VUS).

N	Congenital Heart Disease	Extracardiac Defects	Botto Classification	Type of Genetic Test	Results of Genetic Diagnostics	Genetic Classification
1	ASD, PDA	Partial ACC, feeding difficulties, dysmorphic features, occipital subcutaneous vascular malformation	MCA	CMA+NGS	arr[GRCh38]15q25.2q25.3(85,149,691–85,666,309)×1	VUS
2	CoA, hypoplastic distal aortic arch, BAV, pmVSD, ASD, PDA	hypotonia, hypocalcemia, dysmorphic facies	MCA	CMA	arr[GRCh38]9p21.2(25,713,811–26,334,159)×1	VUS
3	ASD, pmVSD		Isolatated, association	CMA	arr[GRCh38]2q32.3(19,661,4800–196,837,193)×1dn	VUS
					arr[GRCh38]8p23.2(2,470,593–4,801,373)×3 mat	VUS
4 *	ASD, VSD	hypotonia, HCC, moderate ventriculomegaly, dysmorphic facies, hypoplasia of distal phalanx of fifth finger	MCA	CMA+NGS	arr[GRCh38]Xp22.2(14,325,346–14,757,768)×2 mat	VUS
5	CoA, HLHS		MCA	CMA	arr[GRCh38]2q24.2q24.3(163#,517,376–164,167,131)×3 pat	VUS
6	CoA, HLHS	hypotonia		CMA	arr[GRCh38]16q24.1(85,002,354–85,508,509)×1 dn	VUS
7	ToF	coloboma of iris, dysmorphic features	MCA	CMA+NGS	*NOTCH1*(NM_017617.5):c.3190G>A, p.Asp1064Asn	VUS
8	ASD	EA/TEF, annular pancreas, horseshoe kidney, extrarenal pelvis, spina bifida occulta, billiary ducts anomaly	MCA	CMA+NGS	*ZNF462*(NM_021224.6):c.6334C>T, p.Leu2112Phe	VUS
9	CoA	polydactyly, hypospadias, SGA	MCA	CMA+NGS	*TLL1*(NM_012464.5):c.283G>A(pat), p.Gly95Arg	VUS

ACC—agenesis of the corpus callosum, AMC—arthrogryposis multiplex congenita, ASD—atrial septal defect, AVSD—atrioventricular septal defect, BAV—bicuspid aortic valve, CoA—coarctation of aorta, DORV—double-outlet right ventricle, EA/TEF—esophageal atresia/tracheoesophageal fistula, HLHS—hypoplastic left heart syndrome, MCA—multiple congenital anomalies, mVSD—muscular VSD, pat—paternal inheritance, PDA—patent ductus arteriosus, PFO—patent foramen ovale, pmVSD—perimembranous VSD, SGA—small for gestational age, ToF—tetralogy of Fallot, VSD—ventricular septal defect, VUS—variant of unknown significance, * WES trio noninformative.

## Data Availability

The data presented in this study are available on request from the corresponding author for privacy reasons.
